# Retraction Note: Assessment of healthiness among long term inhabiting army soldiers in dry zone of Sri Lanka

**DOI:** 10.1186/s13104-021-05537-4

**Published:** 2021-03-26

**Authors:** Jayaweera Arachchige Asela Sampath Jayaweera, Anpalaham Joseph

**Affiliations:** Department of Microbiology, Faculty of Medicine and Allied Sciences, Rajarata University of Sri Lanka, Anuradhapura, Sri Lanka

## Retraction to: BMC Res Notes (2018) 11:474 https://doi.org/10.1186/s13104-018–3590-4

The Editor has retracted this article [1]. Following publication, concerns were raised regarding the ethics approval for this study. Contrary to the statement in the article, the authors were unable to provide appropriate documentation confirming that ethics approval was obtained for this study. All authors agree to this retraction.
